# CT imaging of gold nanoparticles in a human‐sized phantom

**DOI:** 10.1002/acm2.13155

**Published:** 2021-01-05

**Authors:** Michael Oumano, Liz Russell, Morteza Salehjahromi, Lou Shanshan, Neeharika Sinha, Wilfred Ngwa, Hengyong Yu

**Affiliations:** ^1^ Medical Physics Program Department of Physics and Applied Physics University of Massachusetts Lowell Lowell MA USA; ^2^ Landauer Medical Physics Glenwood IL USA; ^3^ Department of Medical Physics and Radiation Safety Rhode Island Hospital Providence RI USA; ^4^ Neusoft Medical Systems USA Houston TX USA; ^5^ Department of Electrical and Computer Engineering University of Massachusetts Lowell Lowell MA USA; ^6^ Neusoft Medical Systems Hunnan District Shenyang China; ^7^ Department of Physics Wentworth Institute of Technology Boston MA USA; ^8^ Department of Radiation Oncology Brigham and Women's Hospital Boston MA USA

**Keywords:** AuNPs, contrast agents, gold, nanoparticles, x‐rays

## Abstract

**Introduction:**

Gold nanoparticles (AuNPs) are visualized and quantified in a human‐sized phantom with a clinical MDCT scanner.

**Methods:**

Experiments were conducted with AuNPs between 0.00171 and 200 mgAu/mL. CT images were acquired at 80, 100, 120, and 140 kVp in a 33‐cm phantom. Image contrast due to AuNPs was experimentally determined from regions of interest (ROIs) and effective linear attenuation coefficients were calculated from CT x‐ray spectra with consideration of tissue attenuation.

**Results:**

The typical 12‐bit dynamic range of CT images was exceeded for AuNPs at 150 mgAu/mL. A threshold concentration of 0.3–1.4 mgAu/mL was determined for human visualization in 1‐mm images at a typical diagnostic CTDI_vol_ of 23.6 mGy. Optimal image contrast was also achieved at 120 kVp and verified by calculation.

**Conclusions:**

We have shown that scanners capable of reconstructing images with extended Hounsfield scales are required for distinguishing any contrast differences above 150 mgAu/mL. We have also shown that AuNPs result in optimal image contrast at 120 kVp in a human‐sized phantom due to gold’s 80.7 keV k‐edge and the attenuation of x‐rays by tissue. Typical CT contrast agents, like iodine, require the use of lower kVps for optimal visualization, but lower kVps are more difficult to implement in the clinic because of elevated noise levels, elongated scan times, and/or beam‐hardening artifacts. This indicates another significant advantage of AuNPs over iodine not yet discussed in the literature.

## 1 INTRODUCTION

Iodine is the most commonly administered intravenous (IV) contrast agent used today for highlighting specific organs, blood vessels, and/or tissue types. Iodine‐based contrast agents can greatly improve diagnostic accuracy in computed tomography (CT), but they have also been associated with the acute kidney injury (AKI) known as contrast‐induced nephropathy (CIN)^1^ as well as thyroid dysfunction.^2^ Therefore, alternative contrast agents need to be considered. Intense interest was sparked in using gold nanoparticles (AuNPs) as x‐ray/CT contrast agents after the initial reports of Hainfield et al^3^, ^4^ and interest has steadily grown due to several favourable properties of AuNPs.^5^ These properties include a relatively high x‐ray attenuation coefficient, relatively long vascular retention, and the ability to be functionalized.

In addition to the potential diagnostic applications of AuNPs, their therapeutic applications show equally strong promise. At least seven clinical trials are already underway involving AuNPs^6^ and one potential therapeutic application relates to dose enhancement in radiation therapy (RT). Many studies have thus been conducted on AuNPs as x‐ray/CT contrast agents,^7^, ^8^, ^9^, ^10^, ^11^, ^12^, ^13^, ^14^, ^15^, ^16^, ^17^, ^18^, ^19^, ^20^, ^21^, ^22^, ^23^, ^24^, ^25^, ^26^, ^27^, ^28^ but none have yet actually included the visualization or quantification of AuNPs in a human or human‐sized phantom with a clinical multi‐detector CT (MDCT) scanner. This is critical when accounting for clinical kilovolt peak potentials (kVp) in CT imaging, the x‐ray beam filtration used in modern MDCT scanners, the scatter/attenuation that occurs when imaging humans (vs in mice or vials in air), and the detector response of modern MDCT scanners.

We will first establish in this technical note the clinically useful range of concentrations for imaging AuNPs in humans with CT under normal conditions. We will also offer an explanation for the phenomenon that AuNPs result in optimal image contrast at 120 kVp, which is very different from typical CT contrast agents, like iodine, and we will discuss the significant advantage that this offers.

## 2 MATERIALS AND METHODS

### 2.A CT imaging of AuNPs

1.4 nm‐diameter AuNPs were imaged at concentrations below 0.2189 mgAu/mL. These AuNPs were designed and fabricated according to the methods described by Kumar et al.^29^ 15 nm‐diameter AuNPs were also obtained from US Research Nanomaterials, Inc and imaged between 1–200 mgAu/mL. The 15 nm‐diameter AuNPs were first physically suspended in a water‐based acrylic solvent that was dried and hardened. The AuNP‐acrylic mixture was then imaged in the GAMMEX RMI 467 electron density phantom, which has a diameter of 33 cm. A Siemens SOMATOM Definition AS+ was used with the techniques in Table 1.

**Table 1:** Computed tomography scanning parameters.

	Scanning parameters for siemens SOMATOM definition AS+			
	80 kVp	100 kVp	120 kVp	140 kVp
mA	210	210	210	210
Rotation time (s)	1	1	1	1
Pitch	0.6	0.6	0.6	0.6
N (# of detectors)	32	32	32	32
T (detector size in mm)	1.2	1.2	1.2	1.2
N*T = Beam Width (mm)	38.4	38.4	38.4	38.4
SFOV (cm)	50	50	50	50
DFOV (cm)	36	36	36	36
Reconstruction filter	I31s	I31s	I31s	I31s
Iterative reconstruction setting	3 (SAFIRE setting)	3 (SAFIRE setting)	3 (SAFIRE setting)	3 (SAFIRE setting)
CTDI_vol_ (mGy)	6.5	13.8	23.6	36.0
Image slice thickness (mm)	1.0	1.0	1.0	1.0

Computed tomography number contrast was simply calculated by taking the difference between AuNP inserts and adjacent background water using 300 mm^2^ regions of interest (ROIs). The contrast‐to‐noise ratio (CNR) was calculated using Eq. (1)

(1) $$CNR=\frac{HU_{AuNP\;insert}‐HU_{\mathrm{bkg}}}{\sigma_{\mathrm{bkg}}},$$

where HU_AuNP_ insert is the measured CT number for a region of interest (ROI) drawn over AuNP insert, HU_bkg_ is the measured CT number for an ROI drawn over adjacent water background, and σ_bkg_ is the standard deviation for the ROI drawn over the adjacent water background. CNR values were normalized for dose across all kVp stations.

### 2.B Determination of effective linear attenuation coefficients

**Fig. 1:**
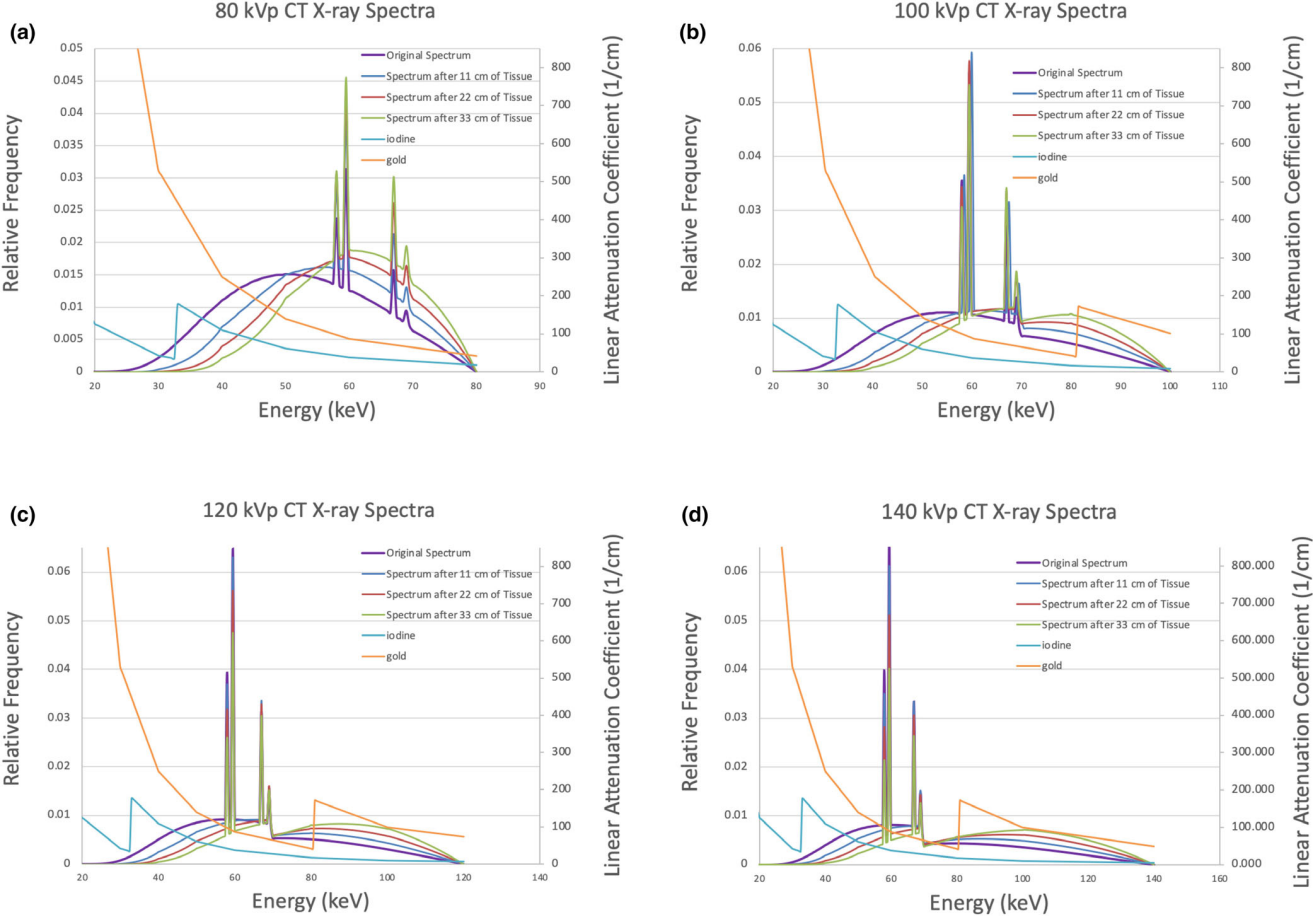
CT x‐ray spectra and effective linear attenuation coefficients of gold and iodine. (a) 80 kVp, (b) 100 kVp, (c) 120 kVp, (d) 140 kVp.

Computed tomography x‐ray spectra were used in conjunction with gold’s mass attenuation coefficient data (Fig. 1), Eq. (2), and the density of gold (19.32 g/cm^3^), to calculate the effective linear attenuation coefficient for the gold nanoparticles

(2) $$\mu_{Au,eff}=\frac{\int_{0}^{E_{\max}}\mu_{\mathrm{Au}}\left(E\right)f\left(E\right)dE}{\int_{0}^{E_{\max}}f\left(E\right)dE},$$

where µ(E) is the energy‐dependent linear attenuation coefficient of gold and f(E) is the photon fluence of the x‐ray spectrum for a given kVp station. The x‐ray spectra were obtained using the IPEM78 program described by Cranley et al.^30^

Theoretically expected linear attenuation coefficients were also qualitatively compared to experimental measurements using the CT images and Eq. (3).

(3) $$HU\left(x,y\right)=1000*\frac{\mu_{\mathrm{eff}}\left(x,y\right)‐\mu_{water,eff}}{\mu_{water,eff}},$$

## 3 RESULTS

### 3.A Image contrast vs kVp

**Fig. 2:**
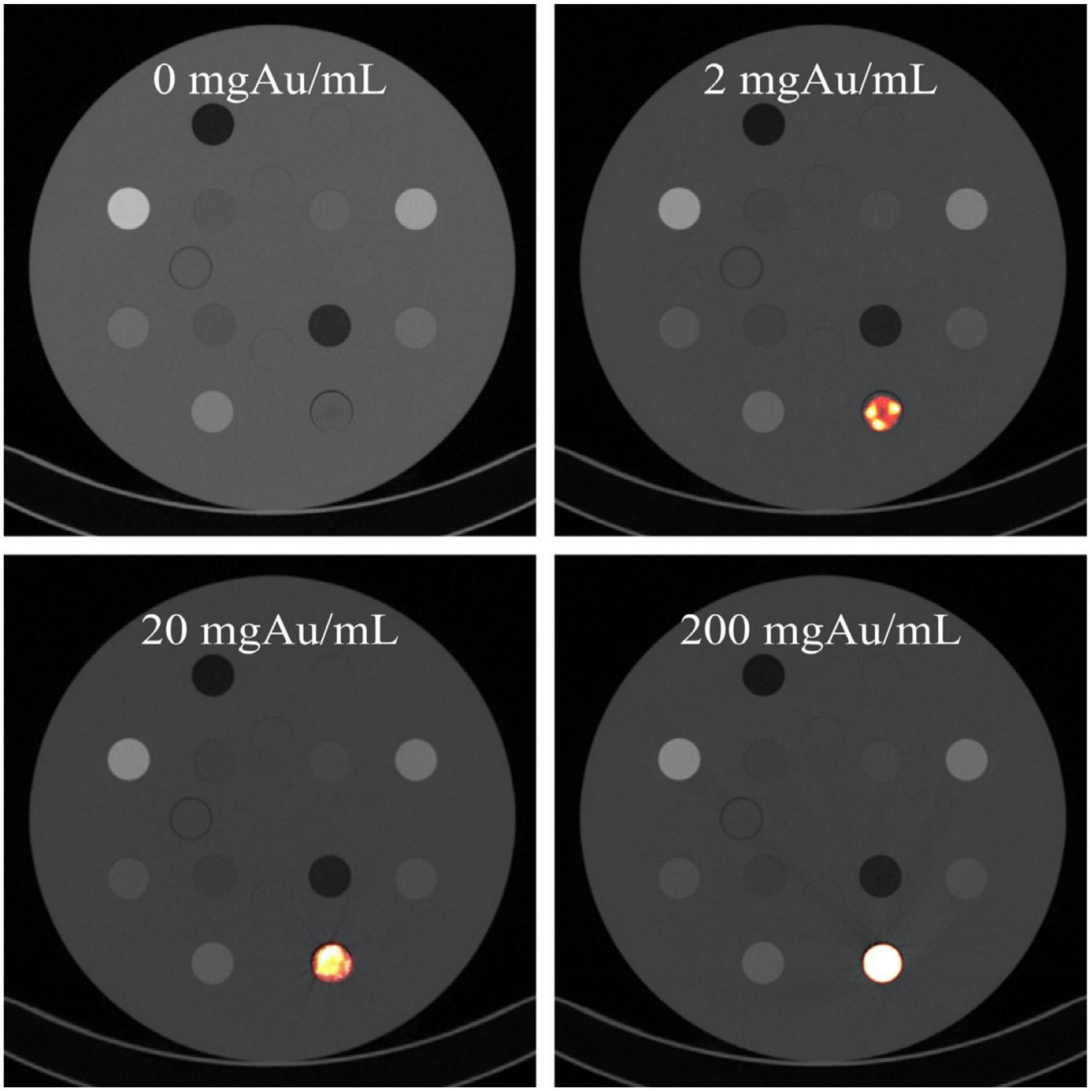
Computed tomography images of AuNPs at 0, 2, 20, and 200 mgAu/mL.

**Fig. 3:**
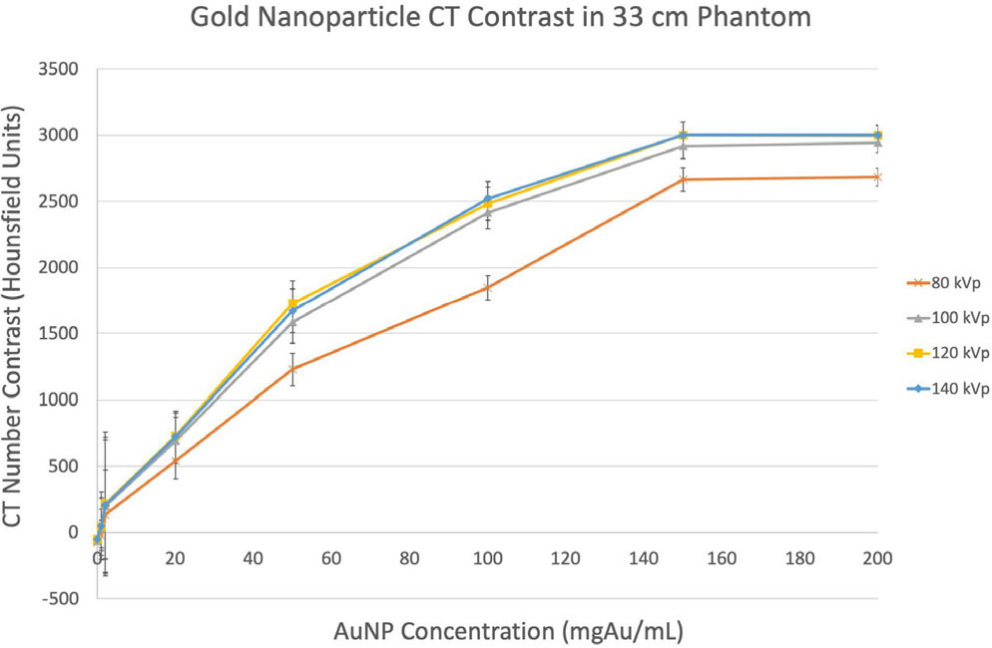
Gold nanoparticle computed tomography contrast in a 33‐cm phantom.

The observed image contrast was generally so extreme in the high‐concentration regime (Figs. 2 and 3), that the typical 12‐bit dynamic range of 4096 (ranging from −1024 to 3072 HU) was exceeded at 150 mgAu/mL and 120 kVp (Fig. 3).

**Fig. 4:**
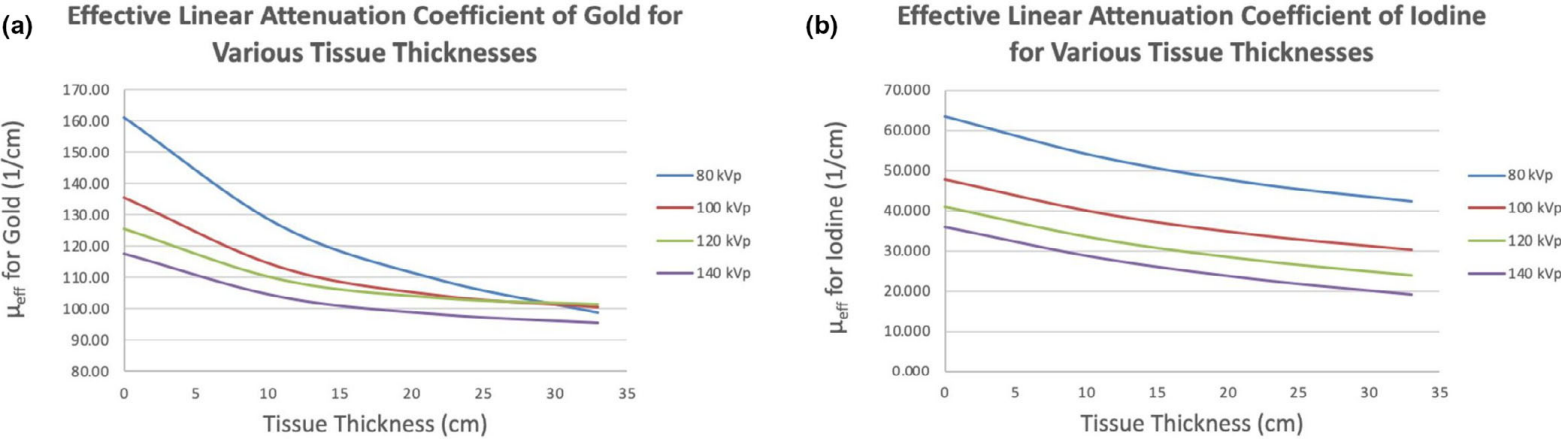
Effective linear attenuation coefficients. (a) gold and (b) iodine.

Typical contrast agents usually require lower kVp settings for optimal image contrast. Iodine’s effective linear attenuation coefficient, for example, is always higher at lower kVps. Because of iodine’s 33 keV k‐edge, this relationship is true despite whether imaging in air or in tissue (Fig. 4). Gold’s 80.7 keV k‐edge, on the other hand, causes the relationship between the effective linear attenuation coefficient and kVp to switch at a given depth in tissue, which we’ve calculated to be 29.4 cm. We have found that gold’s μ_eff_ can be increased by shifting a typical CT scanner’s x‐ray spectrum to lower energies (similar to iodine), but it can also be increased by shifting the spectrum to sit more directly on top of the 80.7 keV k‐edge — as done for higher kVps by beam hardening and tissue attenuation. There are advantages to this that have not yet been discussed in the literature.

### 3.B CNR vs AuNP concentration

**Fig. 5:**
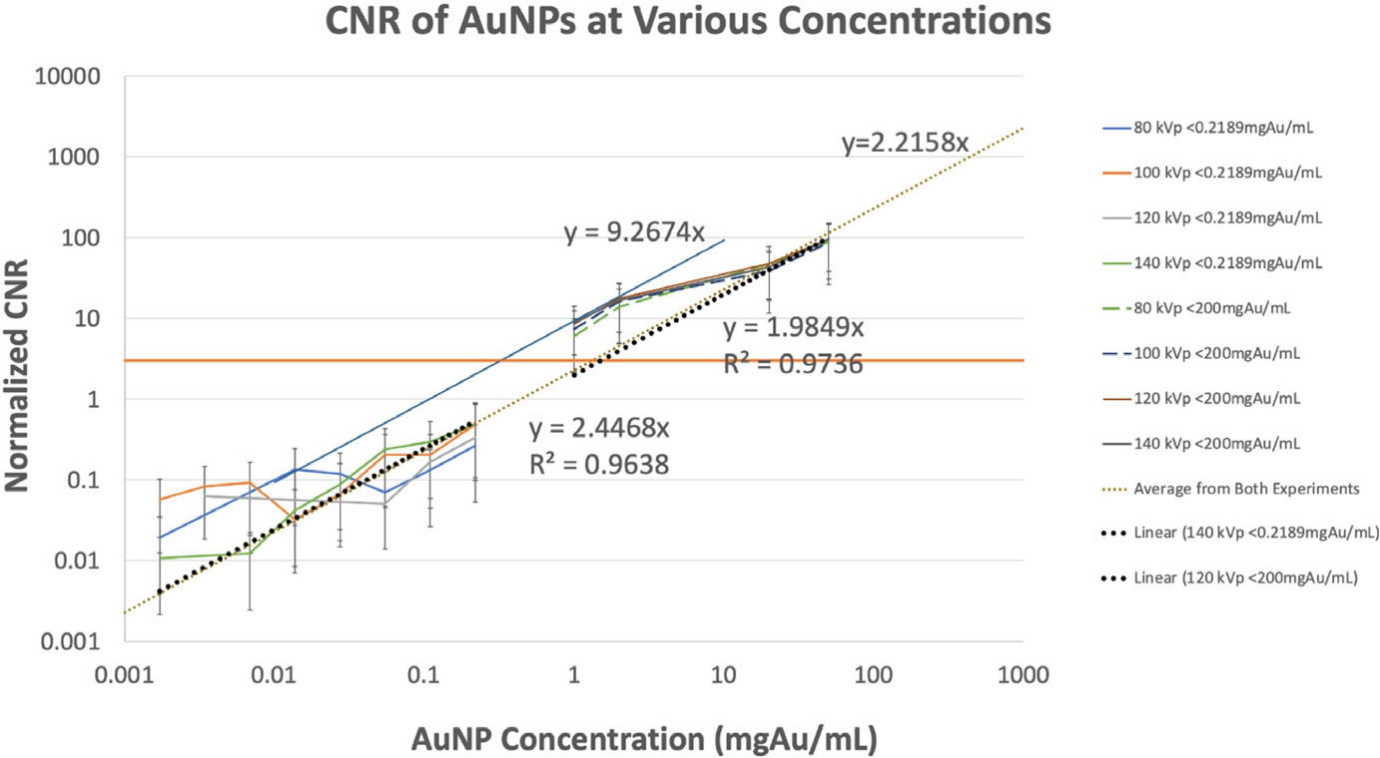
Contrast‐to‐noise ratio quantification of AuNP visibility at various concentrations across approximately 5 orders of magnitude.

All dose‐normalized CNRs for concentrations <0.2189 mgAu/mL were below 0.5 (Fig. 5). Since the Rose Criterion,^31^ states that a CNR of 3–5 is required for a concentration of AuNPs to be visually detectable to an observer, these experiments were successful in establishing a lower limit for visible AuNP concentrations. The CNRs for the four kVp stations ranged from 6 to 9 at 1 mgAu/mL and from 43 to 48 at 20 mgAu/mL. At 200 mgAu/mL, the CNRs ranged from 184 to 209.

Linear interpolation of the low‐ and high‐concentration regimes results in very similar slopes (2.45 and 1.98 (mgAu/mL)^−1^). The average of the two slopes is 2.22 (mgAu/mL)^−1^, which indicates that 1.35 mgAu/mL is required in order to achieve a visually detectable CNR of 3 in 1 mm‐thick images with a typical diagnostic CTDI_vol_ of 23.6 mGy.

Typical slice thicknesses in diagnostic applications are usually 3–5 mm, but considering the important application of treatment planning for radiation therapy, we chose the slice thickness of 1 mm. Just as we have normalized CNRs for dose across kVp stations, one can also normalize for slice thickness by taking the square root of the slice thickness ratio.

The 1 and 2 mgAu/mL data points from the high concentration regime deviate slightly from the linear interpolation. We suspect this is due to the experimental error associated with measuring small quantities of gold nanoparticles and this is supported by dual‐energy CT material decomposition analysis (not discussed in this technical note). Nonetheless, if those two data points are solely interpolated, a slope of 9.27 (mgAu/mL)^−1^ is obtained, which indicates that 0.32 mgAu/mL is required in order to achieve a visually detectable CNR of 3. We thus provide a range of 0.3 to 1.35 mgAu/mL as the required minimum concentration for imaging in humans.

### 3.C Determination of the effective linear attenuation coefficients

The calculated effective linear attenuation coefficients for gold nanoparticles were calculated and graphed above (Fig. 5). These agree with previously published values^32^ and with CT imaging of the AuNPs.

## 4 DISCUSSION

The low concentrations (below 0.2189 mgAu/mL) successfully established a lower limit for imaging AuNPs in humans with clinical MDCT scanners while the high concentrations (1–200 mgAu/mL) successfully established an upper limit. Figure 3 shows a practical upper limit of 150 mgAu/mL since concentrations above this threshold yield absolutely no improvement in contrast unless an extended Hounsfield scale is employed. AuNP clustering is also readily apparent in the high concentration regime below 50 mgAu/mL. ROIs were drawn over these relatively inhomogeneous regions so that average CT number values were averaged over approximately 600 pixels. The reported contrast and CNR values are based on these average values.

Streaking/beam‐hardening artifacts were observed in the immediate vicinity of AuNPs at concentrations of 20 mgAu/mL and increased in severity at higher concentrations. Commercially available iodine‐based agents usually have stock concentrations of about 300 mgI/mL and result in streaking/beam‐hardening artifacts as well — even when diluted. The occurrence of these artifacts depends on many factors such as the reconstruction filter, iterative reconstruction level, and all other attenuating objects within the slice. It is therefore extremely difficult to define a range of concentrations where these artifacts might occur.

Injected solutions will require that AuNPs be in liquid suspension at these concentrations, which we cannot report achieving — even after ultrasonication of the AuNPs. Fortunately, there are reports of liquid suspensions up to 160 mgAu/mL in the literature.^9^

Regarding the size and shape of AuNPs, there have been some attempts to compare these properties, but Ross et al^11^ proved that the size and shape of AuNPs should not have any impact. This was again summarized by Cole in her review article.^5^ We have therefore not addressed any differences between the 1.4 and 15 nm AuNPs.

Figures 3 and 4 do not perfectly agree in that, Fig. 3 shows 120 and 140 kVp to behave almost identically while Fig. 4 indicates that 140 kVp should result in the least image contrast — even in a 33‐cm phantom. This is most likely due to the fact that the x‐ray spectra data used in calculations were specific to a Neusoft NeuViz 128 CT scanner instead of a Siemens SOMATOM Definition AS+. This indicates that different scanners will have slight differences in kVp‐image contrast relationships, but the analysis clearly highlights the impact of gold’s 80.7 keV k‐edge on CT image contrast and perfectly corresponds with measured HUs for 80–120 kVp.

Our finding that the 120 kVp setting results in optimal image contrast in tissue indicates a significant advantage over typical contrast agents like iodine. Iodine requires lower kVps for optimal image contrast, but without adjusting the effective mAs appropriately, elevated noise levels can result in non‐diagnostic images. Adjusting the effective mAs will often require longer scan times due to lower pitch values and/or longer rotation times. This can result in motion artifacts and/or missing the contrast bolus as it travels through blood vessels. In larger patients, lower kVps may be altogether unachievable due to excessive beam hardening. None of these sacrifices need to be made with AuNPs as diagnostic contrast agents.

## 5 CONCLUSION

First, we have shown that AuNPs can be effective contrast agents in diagnostic CT applications. Although the literature is unclear regarding AuNP toxicity, iodine is definitively toxic to certain patients in certain circumstances. AuNPs are also already in the clinic for studies involving laser responsive thermal ablation, plasmonic photothermal therapy, and MRI/US fusion imaging among other applications so it is imperative that we continue exploring the impact of AuNPs on imaging studies as well as their impact on CT imaging in particular.

We have also revealed a significant advantage of AuNPs over typical CT contrast agents, like iodine, in that optimal image contrast is achieved at 120 kVp for average‐sized patients. This means image noise and scan speed need not be sacrificed for optimal image contrast.

## AUTHORS’ CONTRIBUTIONS

Oumano M was responsible for project conceptualization, data analysis, writing of the manuscript, and revisions. Russell L was responsible for data acquisition. Salehjahromi M was responsible for data analysis. Shanshan L was responsible for computer simulations. Sinha N and Ngwa W were responsible for synthesizing AuNPs. Yu H was responsible for project conceptualization, data analysis, writing of the manuscript, and revisions.

## CONFLICT OF INTEREST

No conflict of interest.
